# Poly (Amidehydrazide) Hydrogel Particles for Removal of Cu^2+^ and Cd^2+^ Ions from Water

**DOI:** 10.3390/gels7030121

**Published:** 2021-08-12

**Authors:** Hojung Choi, Taehyoung Kim, Sang Youl Kim

**Affiliations:** Department of Chemistry, Korea Advanced Institute of Science and Technology (KAIST), Daejeon 34141, Korea; ghwnd0214@kaist.ac.kr (H.C.); thkim93@kaist.ac.kr (T.K.)

**Keywords:** copper and cadmium removal, poly (amidehydrazide), hydrogel, hyperbranched polymer, inverse suspension polymerization

## Abstract

Poly(amidoamine)s (PAMAM) are very effective in the removal of heavy metal ions from water due to their abundant amine and amide functional groups, which have a high binding ability to heavy metal ions. We synthesized a new class of hyperbranched poly(amidehydrazide) (PAMH) hydrogel particles from dihydrazides and N,N′-methylenebisacrylamide (MBA) monomer by using the A2 + B4 polycondensation reaction in an inverse suspension polymerization process. In Cd^2+^ and Cu^2+^ ion sorption tests, the synthesized dihydrazide-based PAMH hydrogel particles exhibited sorption capacities of 85 mg/g for copper and 47 mg/g for cadmium. Interestingly, the PAMH showed only a 10% decrease in sorption ability in an acidic condition (pH = 4) compared to the diamine-based hyperbranched PAMAM, which showed a ~90% decrease in sorption ability at pH of 4. In addition, PAMH hydrogel particles remove trace amounts of copper (0.67 ppm) and cadmium (0.5 ppm) in water, below the detection limit.

## 1. Introduction

Water contamination by heavy metal ions has been a long-standing concern since the industrial revolution. Although global attention and efforts to deal with metal contamination have reduced acute heavy metal poisoning accidents, chronic contamination is still ongoing. For example, the usage and disposal of mercury have been constrained since the Minamata accident in 1959, but cases of bioaccumulation of mercury in fish are often found in the oceans [1]. Other examples include the copper contamination of household drinking water in Australia, reported in 2016 [2], and the high level of copper found in the river water of Bangladesh in 2011 [3]. Additionally, cadmium contamination of reservoirs in Poland and of river sediments in India was reported in 2016 [4] and 2018 [5], respectively. As heavy metal contamination remains an ongoing problem, many studies on the removal of heavy metal ions from water have been carried out in recent years [6,7,8,9,10,11,12]. Adsorption is a promising method of removing heavy metal ions from water on the basis of its energy-efficiency in the removal of pollutants [13]. Among the materials that are employed for adsorption, polymer gel adsorbents such as chitosan, alginate, agarose, polyethyleneimine (PEI), and poly(amidoamine) (PAMAM) have been actively investigated due to their high sorption performance, stemming from their functional group-rich nature [14,15,16,17,18,19,20,21,22,23,24,25,26,27]. Among these materials, dendritic PAMAM shows a high sorption capacity because abundant amine and amide groups with dendritic structures facilitate the binding of heavy metal ions. However, the process used to prepare a PAMAM dendrimer is time-consuming. To address this problem, in 2017, our group reported a convenient, one-step method of producing hyperbranched PAMAM hydrogels [28].

Hydrazide is a molecule that has amine and amide groups in its structure, but few cases of its use as an adsorbent have been reported [29,30,31,32,33]. The hydrazide groups are usually introduced into polymer chains with additional steps such as end-group modification [31,32,33].

In this study, we synthesized poly(amidehydrazide) (PAMH) hydrogel particles from dihydrazide and MBA monomers via a one-step A2 + B4 polycondensation method [28,34]. One-pot efficient and economical synthesis is a distinctive advantage of PAMH hydrogels. The synthesis uses a click reaction with an environmentally friendly process that is easy to scale-up. Micrometer-sized hydrogel particles are synthesized by inverse suspension polycondensation through the Aza–Michael addition reaction. This method does not require any additional step to introduce dihydrazide groups into the polymer chains, and the functional group ratio can easily be tuned by adjusting the monomer feed ratio. We carried out Cd^2+^ and Cu^2+^ ion sorption tests with the synthesized dihydrazide-based PAMH hydrogel particles. The polymer exhibited a moderate sorption capacity (~85 mg/g for copper, ~47 mg/g for cadmium), but showed only a 10% decrease in sorption ability in acidic conditions, in contrast with other diamine-based PAMAM polymers (~90% decrease in sorption ability). In addition, trace amounts of copper and cadmium (0.5 ppm) reached levels below the detection limit, indicating the practical applicability of the dihydrazide-based PAMH hydrogels.

## 2. Results and Discussion

**Scheme 1:**
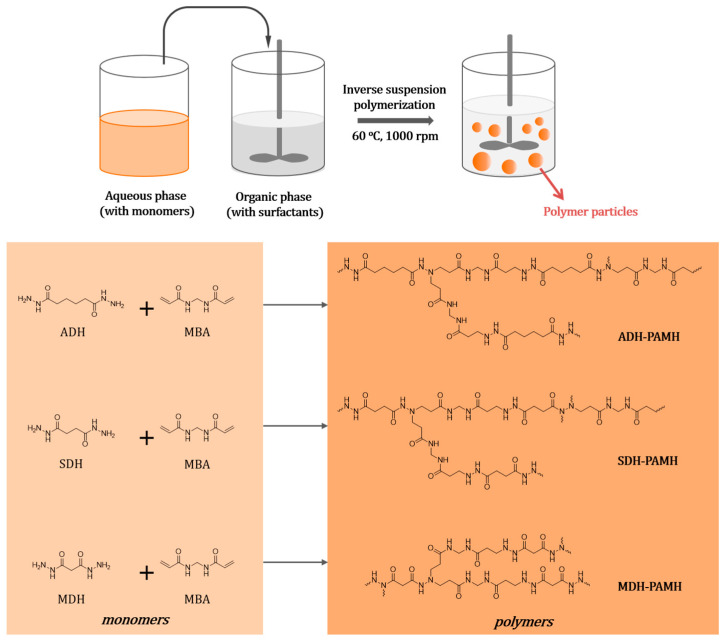
Synthesis of poly(amidehydrazide) (PAMH) particles from adipic acid dihydrazide (ADH), succinic acid dihydrazide (SDH), malonic acid dihydrazide (MDH) and N,N′-methylenebisacrylamide (MBA).

The schematic polymerization process is depicted in Scheme 1. The Aza–Michael addition polymerization of MBA and each dihydrazide monomers was successfully carried out by following the previously reported method [28]. Polycondensation of the B_4_-type amine monomer dihydrazide with A_2_-type acrylamide monomer MBA produces polymer networks. However, in this process, dihydrazide compounds are very soluble in water, while MBA is slightly soluble in water and gradually dissolves into the solution as the reaction proceeds. The solubility difference gives rise to the slow addition of MBA monomer into the solution containing dihydrazide monomers, resulting in the formation of hyperbranched polymers. However, as the polymerization proceeds, the stoichiometry of the two monomers reaches a gel point, where the formation of poly(amidehydrazide) (PAMH) hydrogels occurs. Spherical-shaped PAMH hydrogel particles are obtained by an inverse suspension polymerization process consisting of water and toluene with span 60 as a water-in-oil (W/O) surfactant. The synthesized PAMH polymers are designated as **ADH-PAMH_x_**, **SDH-PAMH_x_**, and **MDH-PAMH_x_**, where x is the feed ratio of MBA to dihydrazide. For example, **ADH-PAMH_1.75_** means [ADH]_0_:[MBA]_0_ = 1:1.75.

**Figure 1:**
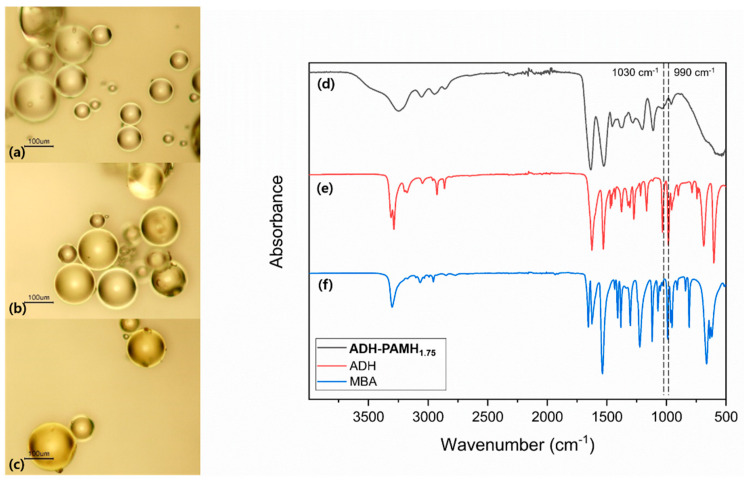
Optical Microscopy (OM) (**a**–**c**) and Attenuated Total Reflection Infrared Spectroscopy (ATR-IR) of **ADH-PAMH_1.75_** (**d**), ADH (**e**), MBA (**f**).

The characterization results of the synthesized polymers with optical microscopy (OM) and Fourier transform attenuated total reflection infrared spectroscopy (ATR-IR) are shown in Figure 1. Spherical shapes are confirmed with optical microscopy (Figure 1a–c), indicating that inverse suspension polymerization proceeded successfully. Particle size showed a range of from 30 μm to 250 μm for all samples (Figure S1). In addition, the synthesized PAMH polymers were analyzed with ATR-IR. Figure 1d–f are representative ATR-IR spectra of **ADH-PAMH_1.75_**, ADH, and MBA. The 1030 cm^−1^ N–N stretching absorption band of hydrazide appears in ADH and **ADH-PAMH_1.75_**, but does not appear in MBA [35]. In addition, the 990 cm^−1^ C=C bending of acrylamide appears in MBA, but does not appear in **ADH-PAMH_1.75_**. The C=C stretching band generally appears at 3050 cm^−1^, but the overtone of amide C=O appears and overlaps with the C=C stretching band at the same range in all samples [34]. The ATR-IR spectra of other synthesized polymers showed the same characteristic absorption bands, indicating that the polymerization proceeded as expected (Figure S2).

**Figure 2:**
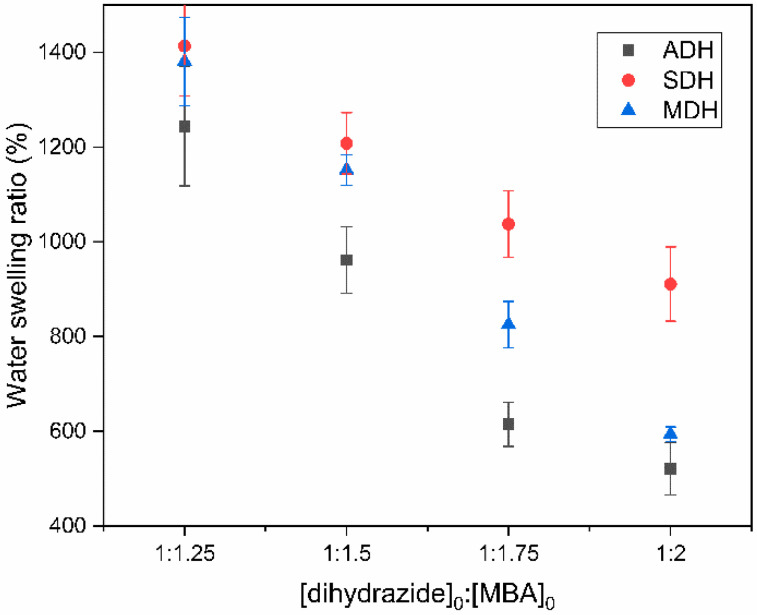
Water-swelling ratio of **ADH-PAMHx** (black, square), **SDH-PAMHx** (red, circle), and **MDH-PAMHx** (blue, triangle).

Before carrying out the heavy metal sorption test, a water-swelling test of PAMH polymer particles was carried out by measuring their weight change after soaking the PAMH polymer for one day in distilled water. Figure 2 shows a decreasing tendency of water swelling by increasing **x** in **dihydrazide-PAMH_x_**. As water uptake has a strong correlation with the crosslinking density of the polymer network, the swelling test results indicate that the crosslinking density of polymer networks becomes greater with an increasing **x** value, as expected. The stoichiometry of the two monomers ranges from one to two (dihydrazide:MBA = 1:2). The polymerization results also support the composition of monomer-repeating units in the polymers that were controlled by the monomer feed ratio. The maximum swelling ratio is 1414% of **SDH-PAMH_1.25_**, reflecting the hydrophilic nature of the PAMH hydrogel particles.

Cu^2+^ sorption ability was measured by following the concentration change in CuCl_2_ aqueous solution after placing dihydrazide-PAMH particles into the solution for 48 h. The concentration of copper metal ions was measured by an inductively coupled plasma optical emission spectrometer (ICP-OES). The copper sorption capacities in the 1000 ppm condition were 74.7 mg/g for **ADH-PAMH_1.75_**, 73.2 mg/g for **SDH-PAMH_1.75_**, and 73.7 mg/g for **MDH-PAMH_1.75_**. To identify the sorption mechanism, Langmuir, Freundlich, and Sips models were applied [10,36,37], and the corresponding R^2^ values were 0.968 (Langmuir), 0.880 (Freundlich), and 0.983 (Sips) for ADH-PAMH_1.75_ (Figure S5). The best fitting was obtained with the Sips equation, which predicts nonuniform surfaces. We believe that the complex structure of hyperbranched hydrogel particles, consisting of amide, terminal primary amines, secondary amines, and tertiary amines at the branching point, is the main reason for this result. Since heavy metal sorption occurs with different functional groups in different chemical environments, the sorption process cannot be predicted solely by the mono-layer adsorption model. Furthermore, to identify moieties involved in the polymer–metal complexation, the ATR-IR of water-swollen **ADH-PAMH_1.75_** and metal-absorbed **ADH-PAMH_1.75_** was measured and compared. Notable shifts were identified in the amine N-H stretching range (3251 cm^−1^) and amine C-N stretching range (1120 cm^−1^), indicating that the main site of complexation (Figure S6). Cd^2+^ sorption ability was measured by following the same process as Cd^2+^ using a CdCl_2_ aqueous solution. The Cd^2+^ sorption capacities in the 1000 ppm condition were 31.2 mg/g for **ADH-PAMH_1.75_**, 47.6 mg/g for **SDH-PAMH_1.75_**, and 41.4 mg/g for **MDH-PAMH_1.75_**. Table 1 shows the sorption capacities of various adsorbents including the PAMH hydrogels synthesized in this study. PAMH hydrogel particles showed moderately comparable sorption capacities compared to the other reported adsorbents. However, PAMH hydrogels show lower Cu^2+^ and Cd^2+^ sorption capacities than the sorption capacities of diamine-based PAMAM particles [28], presumably due to the lower pKa value of the amine group of dihydrazide than that of alkyl amine [38]. It appears that the weak electron-donating power of hydrazide overwhelmed the synergistic CONH-NH_2_ chelating to metal ions. Cu^2+^ sorption tests for the PAMH hydrogel particles with different monomer ratios were also conducted, and representative data of **ADH-PAMH_x_** are shown in Table 2. The Cu^2+^ sorption amount increases with increasing amine content, as expected.

Interestingly, in acidic conditions (pH 4), **ADH-PAMH_1.75_** exhibited only a 10% decrease in Cu^2+^ sorption capacity (63.7 mg/g), while the diamine-based PAMAM hydrogel particles showed a 89% decrease in Cu^2+^ sorption capacity (24.6 mg/g) compared to the sorption capacity at a neutral condition (Figure S4). It appears that the alkyl amines (with a stronger base than hydrazides) are more susceptible to protonation than the hydrazide amines in the acidic condition, and the alkyl amine–metal interactions (coordination) are impeded more than the hydrazide–metal interactions in the acidic condition. In addition, PAMH has more amide groups than PAMAM, but the protonation of amides is less favored than the protonation of amine, and the metal–amide interaction is less affected than the metal–amine interaction at a low pH; this leads the dihydrazide-based PAMH to act as a more effective sorbent than the diamine-based PAMAM in acidic conditions.

The sorption of Cu^2+^ and Cd^2+^ on PAMH was confirmed with an energy dispersive X-ray spectroscopy (EDX) analysis. Metal-ion-absorbed PAMH particles were washed with distilled water, and then dried in a vacuum oven at 50 °C for SEM analysis. In the EDX analysis, 5–15 wt% of copper or cadmium was observed in each sample, even inside the particles. This indicated that the complexation of metal ions occurred not only on the surface but also inside the PAMH hydrogel particles (Figure S3, Table S1). The reusability of particles for heavy metal absorption was investigated with **ADH-PAMH_1.75_**. The desorption of Cu^2+^ was easily achieved through treatment with a 0.1 N HCl solution for 24 h. At a low pH, the chelation between metal ions and nitrogen atoms is weakened, because protons occupy the amine groups. During the recycle steps, the Cu^2+^ absorption capacity declined, but recycled **ADH-PAMH** particles showed about 85% absorption capacity.

**Figure 3:**
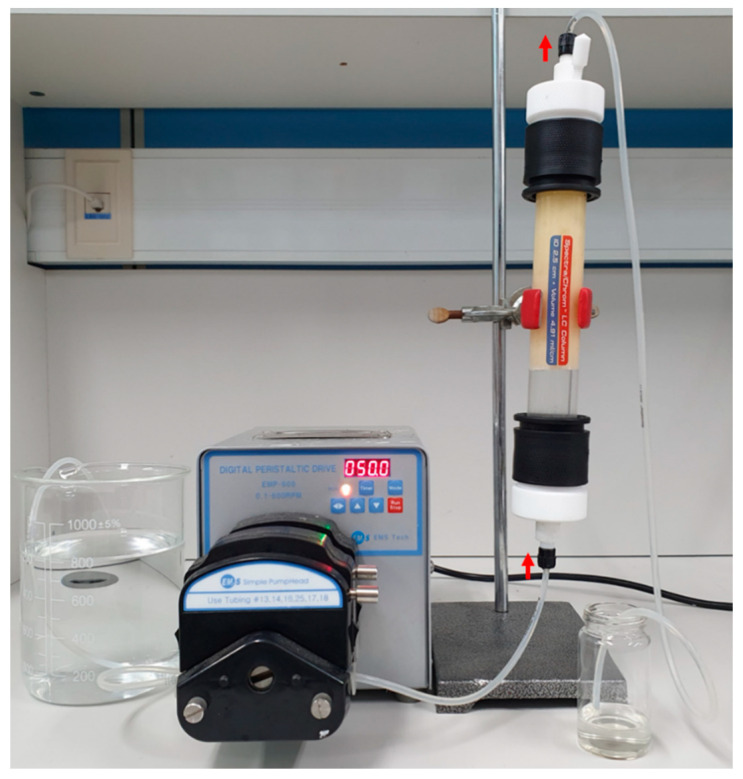
Demonstration of a trace amount of Cu^2+^ and Cd^2+^ sorption test of **ADH-PAMH_1.75_** (flow directions are designated with red arrows).

Copper and cadmium ion removal by the PAMH hydrogel particles (**ADH-PAMH_1.75_**) packed within a glass column was studied with the continuous flow of the solution. The aqueous solutions with 0.67 ppm of Cd^2+^ and 0.5 ppm of Cu^2+^ passed through the packed column (Figure 3). The Cu^2+^ and Cd^2+^ ions were not detected in the solution after passing through the column, indicating that the Cu^2+^ and Cd^2+^ ion concentrations were reduced to below the detection limit of the ICP-OES instrument.

## 3. Conclusions

In this study, we synthesized a new class of hydrogel particles from dihydrazide and MBA monomers via the aza–Michael addition reaction in an inverse suspension polymerization process. This method does not require any additional steps to introduce hydrazide functional groups, unlike other hydrazide-containing adsorbents, and the composition of functional groups can easily be tuned. Cd^2+^ and Cu^2+^ ion sorption studies showed the polymer exhibited a tunable sorption ability depending on the monomer ratio, and the maximum sorption capacity was 85 mg/g for Cu^2+^ and 47.6 mg/g for Cd^2+^, which are comparable values to other reported adsorbents. The polymer showed a 10% decrease in sorption ability in acidic conditions, unlike another PAMAM polymer, which showed a ~90% decrease in sorption ability at a pH of 4, suggesting high sorption performance in acidic conditions.

## 4. Materials and Methods

### 4.1. Materials

Adipic acid dihydrazide (ADH), succinic acid dihydrazide (SDH), malonic acid dihydrazide (MDH), N,N′-methylenebis (acrylamide), Span 60^®^, CuCl_2_ anhydrous, CdCl_2_ were purchased from Sigma-Aldrich (Boston, MA, USA), Toluene was purchased from Junsei (Tokyo, Japan). All reactants were used without further purification. Optical microscopy (OM) images were obtained on a Nikon Eclipse (ME600) (Tokyo, Japan) and field emission scanning electron microscopy (FE-SEM) images were obtained from Hitachi SU 8230 (Tokyo, Japan). Energy-dispersive X-ray analysis (EDX) was performed on a Hitachi SU 8230 (Tokyo, Japan). Metal ion concentration was measured by an inductively coupled plasma optical emission spectrometer (Agilent ICP-OES 5110, Santa Clara, CA, USA). Fourier transform attenuated total reflectance infrared spectroscopy (FT ATR-IR) spectra were obtained from a Nicolet iS50 (Thermo Scientific, Hampton, NH, USA).

### 4.2. Methods

#### 4.2.1. Synthesis of Poly(Amidehydrazide) Particles

An oil phase was prepared in a 50 mL round bottom flask as the suspension stabilizer span 60 (1.0 wt% of the monomers) was dissolved in toluene (12 mL) and heated to 60 °C with vigorous agitation. Methylenebis(acrylamide) (MBA) was placed in another 50 mL round-bottom flask with aqueous NaOH solution (6 mL) to form an aqueous phase, and heated to 50 °C. A dihydrazide monomer (ADH, SDH, MDH) was added to the MBA solution. The aqueous mixture was heated at 50 °C for 5–10 min until the two monomers completely dissolved. After the aqueous solution became transparent, it was poured into the oil phase solution and then agitated at 1000 rpm at 60 °C for 12 h to conduct inverse suspension polymerization. After the polymerization, off-white polymer particles were produced, and these particles were filtered and washed several times with distilled water, acetone and methanol. The particles were dried at 60 °C with reduced pressure overnight.

#### 4.2.2. Measurement of Metal Ion Sorption Test

Stock copper solution was prepared with the initial concentration of copper (C_i_) in deionized water using CuCl_2_. Dry polymer particles were placed in the copper solution and kept for 24 h to reach equilibrium at room temperature. Copper-absorbed polymer particles were removed by a 0.45 μm Nylon syringe filter, and the filtrate was collected. The concentration of copper in the filtrate (C_f_) was determined using an Inductively Coupled Plasma Optical Emission Spectrometer (Agilent ICP-OES 5110, Santa Clara, CA, USA). Copper ion absorption capacity (A) was calculated from Equation (1) below. Sorption isotherms were fitted following Equations (2)–(4).

Copper ion absorption capacity:

(A) = [(C_i_ − C_f_)V/m] (g/g) (1)

where m (mg) is the weight of the dry sample and V (mL) is the volume of the stock copper solution. C_i_ and C_f_ (mg/mL) are the initial and filtrate copper ion concentrations, respectively. The same measuring process was conducted for the cadmium sorption test.

Langmuir equation:

(2) $$\frac{C_{e}}{q_{e}}=\frac{1}{q_{m}K_{L}}+{\;C}_{e}\left(\frac{1}{q_{m}}\right)$$

C_e_, the equilibrium concentration (mg/L), q_e_, equilibrium adsorption capacity (mg/g), q_m_ (mg/g), and K_L_ (L/mg) are, respectively, the capacity and binding energy of adsorption.

Freundlich equation:

(3) $$\log q_{e}\;={\;\mathrm{logK}}_{F}\;+\;\frac{1}{n}\log C_{e}$$

C_e_, the equilibrium concentration (mg/L), q_e_, equilibrium adsorption capacity (mg/g), and K_F_ and n are Freundlich constants related to the adsorption capacity and adsorption intensity, respectively.

Sips equation:

(4) $$q_{e}\;=\;\frac{q_{\mathrm{sat}}{\mathrm{KC}}_{e}^{n}{}^{}}{1\;+{\;\mathrm{KC}}_{e}^{n}}$$

C_e_, the equilibrium concentration (mg/L), q_e_, equilibrium adsorption capacity (mg/g), and q_sat,_ K, and n are Sips maximum adsorption capacity (mg/g), Sips equilibrium constant, and heterogeneity factor, respectively.

#### 4.2.3. Measurement of Trace Amount Metal Ion Sorption Test (Packed Column Method)

A stock copper solution of 0.5 ppm was prepared with initial concentration of copper (C_i_) in deionized water using CuCl_2_. Dry polymer particles (~20 g) were packed in a Spectra/chrom^®^ LC column (Fisher Scientific, Hampton, NH, USA) and the copper solution flowed through the column at 5 mL/min by a digital peristaltic drive EMP-600 (EMS tech, Yongin, Korea). The eluates were collected every 5 min for one hour at room temperature. Copper absorbed polymer particles were removed by a 0.45 μm Nylon syringe filter, and the filtrate was collected. The concentration of copper in the eluate was determined by Inductively Coupled Plasma Optical Emission Spectrometer (Agilent ICP-OES 5110, Santa Clara, CA, USA). The same measuring process was conducted for the cadmium sorption test.

#### 4.2.4. ATR-IR of Water-Swollen and Metal-Absorbed **ADH-PAMH_1.75_**

To mimic the real conditions, water-swollen wet particles were compared with metal-absorbed particles. Dried **ADH-PAMH_1.75_** particles (20 mg) were soaked for 24 h in 10 mL of distilled water, 1000 ppm Cu^2+^ solution, and 1000 ppm Cd^2+^ solution, respectively. The polymer particles were then filtered and washed with distilled water to remove residual metal ions. ATR-IR spectroscopy was measured with Nicolet iS50 (Thermo Scientific, Hampton, NH, USA) for the filtered polymer particles before the particles dried.

## Figures and Tables

**Table 1:** Cu(II) ion sorption abilities of various adsorbents.

Heavy Metal Ion	Adsorbents	Sorption Capacity (mg/g)	References
Cu^2+^	Activated carbon	23.0	[39]
	Bacteria isolated from soil	16.3	[40]
	Dried sunflower leaves	89.4	[41]
	Poly (amide-hydrazide-imide)s containing L-aspartic acid	34.3	[42]
	Aniline grafted chitosan	106.6	[43]
	PVDF-g-PAA-PAMAM membranes	101.0	[44]
	Cellulose nanofibril-polyethyleneimine	52.3	[45]
	Lignite	21.4	[46]
	**ADH-PAMH_1.25_**	85.3	This work
Cd^2+^	Activated carbon	33.6	[47]
	Alginate beads	31.4	[48]
	Alginate-chitosan composite	90.8	[49]
	HEMA-PGMA functionalized DETA	35.9	[50]
	Fe_3_O_4_@APS@AA-co-CA MNPs	29.6	[51]
	Poly (amide-hydrazide-imide)s containing L-aspartic acid	79.6	[42]
	Lignite	38.0	[46]
	**SDH-PAMH_1.25_**	47.6	This work

**Table 2:** Amine, amide compositions, and Cu^2+^ sorption amount at 1000 ppm Cu^2+^ solution of **ADH-PAMH_x_**.

	Amine (g/g)	Amide (g/g)	Cu^2+^ Sorption at 1000 ppm Solution (mg/g)
**ADH-PAMH_1.25_**	0.087	0.528	85.27
**ADH-PAMH_1.5_**	0.079	0.531	84.34
**ADH-PAMH_1.75_**	0.072	0.533	74.65
**ADH-PAMH_2_**	0.066	0.535	71.87

## Data Availability

The data presented in this study are available on request from the corresponding author.

## Supplementary Materials

The following are available online at https://www.mdpi.com/article/10.3390/gels7030121/s1, Figure S1: OM images of **dihydrazide-PAMH_x_**, Figure S2: ATR-IR spectra of **SDH-PAMH_1.75_** and **MDH-PAMH_1.75_**, Figure S3: SEM images of **dihydrazide-PAMH_1.75_** for Cu^2+^ sorption and Cd^2+^ sorption, Figure S4: Cu^2+^ sorption test in acidic condition (pH 4) and neutral condition. Figure S5: Cu^2+^ Sorption isotherm of **ADH-PAMH_1.75_**, **SDH-PAMH_1.75_**, and **MDH-PAMH_1.75_**. Figure S6: ATR-IR for metal absorbed **ADH-PAMH_1.75_** (black: wet **ADH-PAMH_1.75_**, red: Cu^2+^ absorbed **ADH-PAMH_1.75_**, blue: Cd^2+^ absorbed **ADH-PAMH_1.75_**). Table S1: EDX data of **ADH-PAMH_1.75_**, **SDH-PAMH_1.75_**, and **MDH-PAMH_1.75_**. All values of table are weight percent (%).

## References

[1] Peterson S.A., Van Sickle J., Herlihy A.T., Hughes R.M. (2007). Mercury concentration in fish from streams and rivers throughout the western United States. Environ. Sci. Technol..

[2] Harvey P.J., Handley H.K., Taylor M.P. (2016). Widespread copper and lead contamination of household drinking water, New South Wales, Australia. Environ. Res..

[3] Mohiuddin K.M., Ogawa Y., Zakir H.M., Otomo K., Shikazono N. (2011). Heavy metals contamination in water and sediments of an urban river in a developing country. Int. J. Environ. Sci. Technol..

[4] Rzętała M.A. (2016). Cadmium contamination of sediments in the water reservoirs in Silesian Upland (southern Poland). J. Soils Sediments.

[5] Idrees N., Tabassum B., Abd Allah E.F., Hashem A., Sarah R., Hashim M. (2018). Groundwater contamination with cadmium concentrations in some West, U.P. Regions, India. Saudi J. Biol. Sci..

[6] He H., Lu Q., Huang H., Xue F., Lin W., Zhou H., Wei W. (2020). Biomass bagasse-based hyperbranched adsorbent for the complete removal of low-level Cr(VI). Cellulose.

[7] Falahian Z., Torki F., Faghihian H. (2018). Synthesis and Application of Polypyrrole/Fe 3 O 4 Nanosize Magnetic Adsorbent for Efficient Separation of Hg 2+ from Aqueous Solution. Glob. Challenges.

[8] Choi H.Y., Bae J.H., Hasegawa Y., An S., Kim I.S., Lee H., Kim M. (2020). Thiol-functionalized cellulose nanofiber membranes for the effective adsorption of heavy metal ions in water. Carbohydr. Polym..

[9] Wu Q., He H., Zhou H., Xue F., Zhu H., Zhou S., Wang L., Wang S. (2020). Multiple active sites cellulose-based adsorbent for the removal of low-level Cu(II), Pb(II) and Cr(VI) via multiple cooperative mechanisms. Carbohydr. Polym..

[10] Huang Y., Wu H., Shao T., Zhao X., Peng H., Gong Y., Wan H. (2018). Enhanced copper adsorption by DTPA-chitosan/alginate composite beads: Mechanism and application in simulated electroplating wastewater. Chem. Eng. J..

[11] Rahman M.L., Fui C.J., Ting T.X., Sarjadi M.S., Arshad S.E., Musta B. (2020). Polymer ligands derived from jute fiber for heavy metal removal from electroplating wastewater. Polymers.

[12] Azizkhani S., Mahmoudi E., Abdullah N., Ismail M.H.S., Mohammad A.W., Hussain S.A. (2020). Synthesis and characterisation of graphene oxide-silica-chitosan for eliminating the Pb(II) from aqueous solution. Polymers.

[13] Fernández-González R., Martín-Lara M.A., Moreno J.A., Blázquez G., Calero M. (2019). Effective removal of zinc from industrial plating wastewater using hydrolyzed olive cake: Scale-up and preparation of zinc-Based biochar. J. Clean. Prod..

[14] Diallo M.S., Christie S., Swaminathan P., Johnson J.H., Goddard W.A. (2005). Dendrimer enhanced ultrafiltration. 1. Recovery of Cu(II) from aqueous solutions using PAMAM dendrimers with ethylene diamine core and terminal NH 2 groups. Environ. Sci. Technol..

[15] Cahill B.P., Papastavrou G., Koper G.J.M., Borkovec M. (2008). Adsorption of poly(amido amine) (PAMAM) dendrimers on silica: Importance of electrostatic three-body attraction. Langmuir.

[16] Gosika M., Maiti P.K. (2018). PH and generation dependent morphologies of PAMAM dendrimers on a graphene substrate. Soft Matter.

[17] Ren B., Wang K., Zhang B., Li H., Niu Y., Chen H., Yang Z., Li X., Zhang H. (2019). Adsorption behavior of PAMAM dendrimers functionalized silica for Cd(II) from aqueous solution: Experimental and theoretical calculation. J. Taiwan Inst. Chem. Eng..

[18] Sohail I., Bhatti I.A., Ashar A., Sarim F.M., Mohsin M., Naveed R., Yasir M., Iqbal M., Nazir A. (2020). Polyamidoamine (PAMAM) dendrimers synthesis, characterization and adsorptive removal of nickel ions from aqueous solution. J. Mater. Res. Technol..

[19] Zhang X., Qin Y., Zhang G., Zhao Y., Lv C., Liu X., Chen L. (2019). Preparation of PVDF/hyperbranched-nano- palygorskite composite membrane for efficient removal of heavy metal ions. Polymers.

[20] Abdelwahab H.E., Hassan S.Y., Mostafa M.A., El Sadek M.M. (2016). Synthesis and characterization of glutamic-chitosan hydrogel for copper and nickel removal from wastewater. Molecules.

[21] Paraskevopoulou P., Raptopoulos G., Leontaridou F., Papastergiou M., Sakellari A., Karavoltsos S. (2021). Evaluation of polyurea-crosslinked alginate aerogels for seawater decontamination. Gels.

[22] Chen Y., Chen L., Bai H., Li L. (2013). Graphene oxide-chitosan composite hydrogels as broad-spectrum adsorbents for water purification. J. Mater. Chem. A.

[23] Bessbousse H., Rhlalou T., Verchère J.F., Lebrun L. (2008). Removal of heavy metal ions from aqueous solutions by filtration with a novel complexing membrane containing poly(ethyleneimine) in a poly(vinyl alcohol) matrix. J. Memb. Sci..

[24] Verma A., Thakur S., Mamba G., Gupta R.K., Thakur P., Thakur V.K. (2020). Graphite modified sodium alginate hydrogel composite for efficient removal of malachite green dye. Int. J. Biol. Macromol..

[25] Sansonetti A., Bertasa M., Corti C., Rampazzi L., Monticelli D., Scalarone D., Sassella A., Canevali C. (2021). Optimization of Copper Stain Removal from Marble through the Formation of Cu (II) Complexes in Agar Gels. Gels.

[26] Thakur S., Sharma B., Verma A., Chaudhary J., Tamulevicius S., Thakur V.K. (2018). Recent progress in sodium alginate based sustainable hydrogels for environmental applications. J. Clean. Prod..

[27] Sharma B., Thakur S., Mamba G., Gupta R.K., Gupta V.K., Thakur V.K. (2021). Titania modified gum tragacanth based hydrogel nanocomposite for water remediation. J. Environ. Chem. Eng..

[28] Lee S., Eom Y., Park J., Lee J., Kim S.Y. (2017). Micro-hydrogel Particles Consisting of Hyperbranched Polyamidoamine for the Removal of Heavy Metal Ions from Water. Sci. Rep..

[29] Okesola B.O., Suravaram S.K., Parkin A., Smith D.K. (2016). Selective Extraction and In Situ Reduction of Precious Metal Salts from Model Waste To Generate Hybrid Gels with Embedded Electrocatalytic Nanoparticles. Angew. Chem..

[30] Okesola B.O., Smith D.K. (2013). Versatile supramolecular pH-tolerant hydrogels which demonstrate pH-dependent selective adsorption of dyes from aqueous solution. Chem. Commun..

[31] bt Johari I.S., Yusof N.A., Haron M.J., Mohd Nor S.M. (2013). Preparation and characterization of poly(ethyl hydrazide) grafted oil palm empty fruit bunch for removal of ni(ii) ion in aqueous environment. Polymers.

[32] Hamza M.F., Aly M.M., Abdel-Rahman A.A.H., Ramadan S., Raslan H., Wang S., Vincent T., Guibal E. (2017). Functionalization of magnetic chitosan particles for the sorption of U(VI), Cu(II) and Zn(II)-hydrazide derivative of glycine-grafted chitosan. Materials.

[33] Park S.H., Shin S.S., Park C.H., Jeon S., Gwon J., Lee S.Y., Kim S.J., Kim H.J., Lee J.H. (2020). Poly(acryloyl hydrazide)-grafted cellulose nanocrystal adsorbents with an excellent Cr(VI) adsorption capacity. J. Hazard. Mater..

[34] Choi H., Eom Y., Lee S., Kim S.Y. (2019). Copper Ions Removal from Water using A2B3 Type Hyperbranched Poly(amidoamine) Hydrogel Particles. Molecules.

[35] Dirtu D., Odochian L., Pui A., Humelnicu I. (2006). Thermal decomposition of ammonia. N2H4-An intermediate reaction product. Cent. Eur. J. Chem..

[36] Tzabar N., ter Brake H.J.M. (2016). Adsorption isotherms and Sips models of nitrogen, methane, ethane, and propane on commercial activated carbons and polyvinylidene chloride. Adsorption.

[37] Huang W., Diao K., Tan X., Lei F., Jiang J., Goodman B.A., Ma Y., Liu S. (2019). Mechanisms of adsorption of heavy metal cations from waters by an amino bio-based resin derived from Rosin. Polymers.

[38] Raddatz S., Mueller-Ibeler J., Kluge J., Wäß L., Burdinski G., Havens J.R., Onofrey T.J., Wang D., Schweitzer M. (2002). Hydrazide oligonucleotides: New chemical modification for chip array attachment and conjugation. Nucleic Acids Res..

[39] Yuan Y., Zhang G., Li Y., Zhang G., Zhang F., Fan X. (2013). Poly(amidoamine) modified graphene oxide as an efficient adsorbent for heavy metal ions. Polym. Chem..

[40] Li Q., Bai Q., Xu F., Zhang Q., Chu S. Absorptive Removal of Copper from Aqueous Solution by Biosorbents. Proceedings of the 2011 International Conference on Chemistry and Chemical Process.

[41] Benaïssa H., Elouchdi M.A. (2007). Removal of copper ions from aqueous solutions by dried sunflower leaves. Chem. Eng. Process. Process. Intensif..

[42] Vakili M.R., Zahmatkesh S., Panahiyan M.J., Jafarizadeh T. (2015). Poly(amide-hydrazide-imide)s containing L-aspartic acid: Synthesis, characterization, and their applications in removal of heavy metal ions. Des. Monomers Polym..

[43] Bahareh V.D.W. (2020). Cu(II) Ion Adsorption by Aniline Grafted Chitosan and Its Responsive Fluorescence Properties. Molecules.

[44] Sun H., Zhang X., He Y., Zhang D., Feng X., Zhao Y., Chen L. (2019). Preparation of PVDF-g-PAA-PAMAM membrane for efficient removal of copper ions. Chem. Eng. Sci..

[45] Zhang N., Zang G.L., Shi C., Yu H.Q., Sheng G.P. (2016). A novel adsorbent TEMPO-mediated oxidized cellulose nanofibrils modified with PEI: Preparation, characterization, and application for Cu(II) removal. J. Hazard. Mater..

[46] Jellali S., Azzaz A.A., Jeguirim M., Hamdi H., Mlayah A. (2021). Use of lignite as a low-cost material for cadmium and copper removal from aqueous solutions: Assessment of adsorption characteristics and exploration of involved mechanisms. Water.

[47] Kobya M., Demirbas E., Senturk E., Ince M. (2005). Adsorption of heavy metal ions from aqueous solutions by activated carbon prepared from apricot stone. Bioresour. Technol..

[48] Mata Y.N., Blázquez M.L., Ballester A., González F., Muñoz J.A. (2009). Biosorption of cadmium, lead and copper with calcium alginate xerogels and immobilized Fucus vesiculosus. J. Hazard. Mater..

[49] Hamza M.F., Hamad N.A., Hamad D.M., Khalafalla M.S., Abdel-rahman A.A., Zeid I.F., Wei Y., Hessien M.M., Fouda A., Salem W.M. (2021). Synthesis of Eco-Friendly Biopolymer, Alginate-Chitosan Composite to Adsorb the Heavy Metals, Cd(II) and Pb(II) from Contaminated Effluents. Materials.

[50] Trakulsujaritchok T., Noiphom N., Tangtreamjitmun N., Saeeng R. (2011). Adsorptive features of poly(glycidyl methacrylate-co-hydroxyethyl methacrylate): Effect of porogen formulation on heavy metal ion adsorption. J. Mater. Sci..

[51] Ge F., Li M.M., Ye H., Zhao B.X. (2012). Effective removal of heavy metal ions Cd 2+, Zn 2+, Pb 2+, Cu 2+ from aqueous solution by polymer-modified magnetic nanoparticles. J. Hazard. Mater..

