# Supplementary material for: Poly (Amidehydrazide) Hydrogel Particles for Removal of Cu2+ and Cd2+ Ions from Water
Source: Gels. 2021 Aug 12;7(3):121. doi: 10.3390/gels7030121 (PMC8395747; doi:10.3390/gels7030121)
Supplement: Supplementary file 1 [file gels-07-00121-s001.zip › gels-1307580-SI.pdf]

gels

MDPI

Communication

Poly (Amidehydrazide) Hydrogel Particles for Removal of Cu^{2+} and Cd^{2+} Ions from Water

Hojung Choi, Taehyoung Kim and Sang Youl Kim *

Supplementary Materials:

1. Optical Microscope (OM) images of ADH-PAMH$_x$, SDH-PAMH$_x$ and MDH-PAMH$_x$

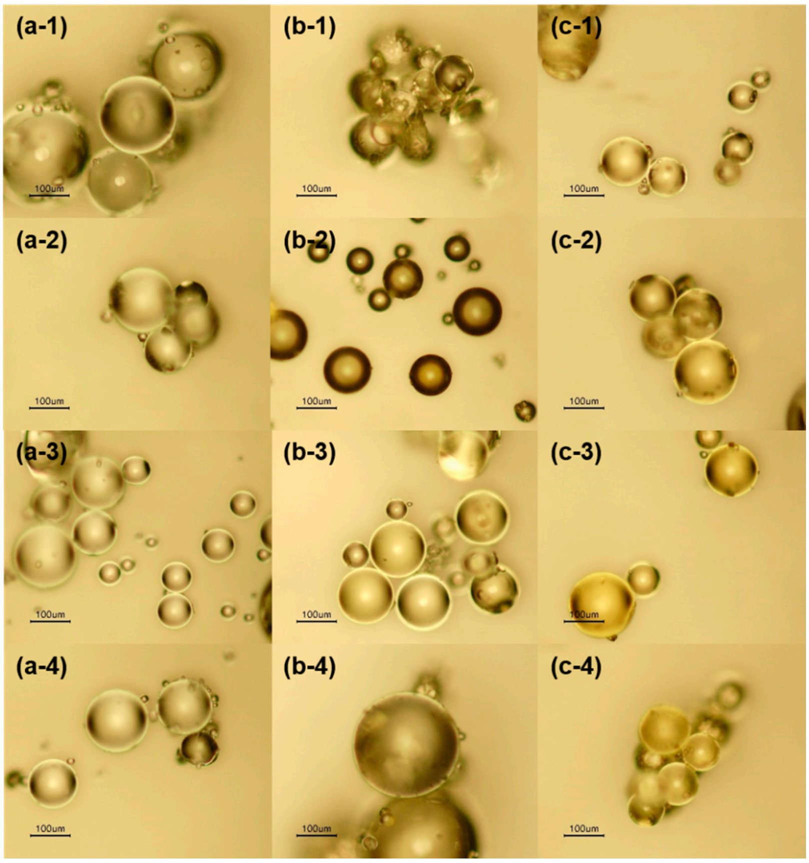

Figure S1. OM images of ADH-PAMH$_x$ (a), SDH-PAMH$_x$ (b), MDH-PAMH$_x$ (c) for different x of 1.25 (1), 1.5 (2), 1.75 (3), 2 (4). For example, (b-2) is an image of SDH-PAMH$_{1.5}$.

2. ATR-IR spectra of SDH-PAMH$_x$ and MDH-PAMH$_x$

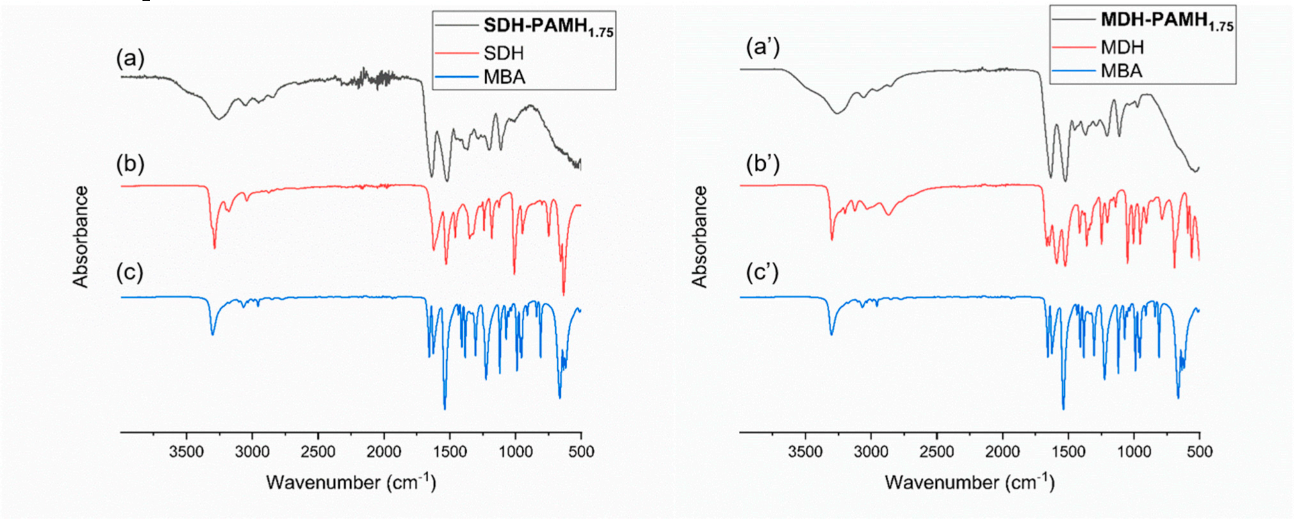

Figure S2. ATR-IR spectra of **SDH-PAMH$_{1.75}$** (a), SDH (b), MBA (c), **MDH-PAMH$_{1.75}$** (a'), MDH (b'), MBA (c'). .

3. Scanning Electron Microscopy (SEM) images and EDX analysis of ADH-PAMH$_{1.75}$, SDH-PAMH$_{1.75}$ and MDH-PAMH$_{1.75}$

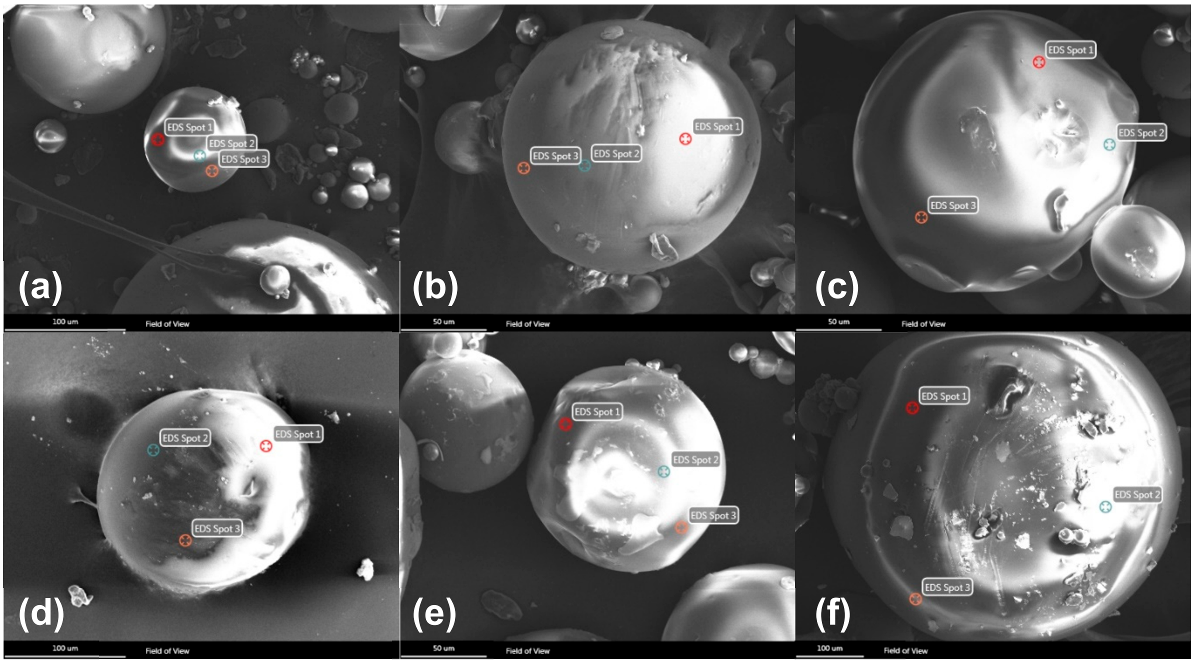

Figure S3. SEM images of **ADH-PAMH$_{1.75}$ (a,d)**, **SDH-PAMH$_{1.75}$ (b,e)**, **MDH-PAMH$_{1.75}$ (c,f)** for Cu^{2+} sorption **(a,b,c)** and Cd^{2+} sorption **(d,e,f)**.

Table S1. EDX data of **ADH-PAMH$_{1.75}$, SDH-PAMH$_{1.75}$,** and **MDH-PAMH$_{1.75}$.** all values of table are weight percent (%).

**Element**	**ADH-PAMH$_{1.75}$**		**SDH-PAMH$_{1.75}$**		**MDH-PAMH$_{1.75}$**	
	**Cu^{2+}**	**Cd^{2+}**	**Cu^{2+}**	**Cd^{2+}**	**Cu^{2+}**	**Cd^{2+}**
C K	45.43	55.94	38.97	47.43	39.52	47.34
N K	19.70	22.21	18.63	23.02	26.94	18.24
O K	14.89	16.08	14.64	14.48	17.68	23.95
Cu L	10.38	-	13.43	-	10.70	-
Cd L	-	3.84	-	10.34	-	7.67
Cl K	9.59	1.93	14.32	4.73	5.15	2.81

4. Cu^{2+} sorption test in acidic condition

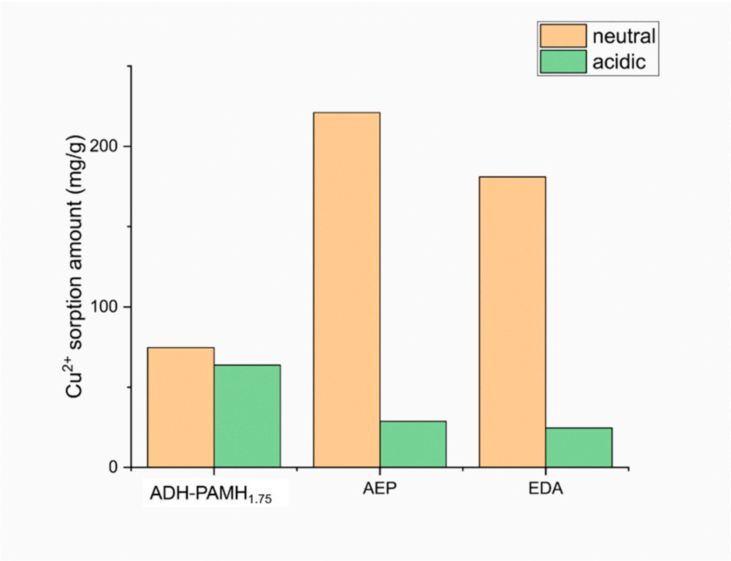

Figure S4. Cu^{2+} sorption test in acidic condition (pH 4) and neutral condition. AEP is PAMAM gel consist of aminoethylpiperazine and MBA, EDA is PAMAM gel consist of ethylenediamine and MBA.

5. Cu^{2+} sorption isotherm of dihydrazide-PAMHx

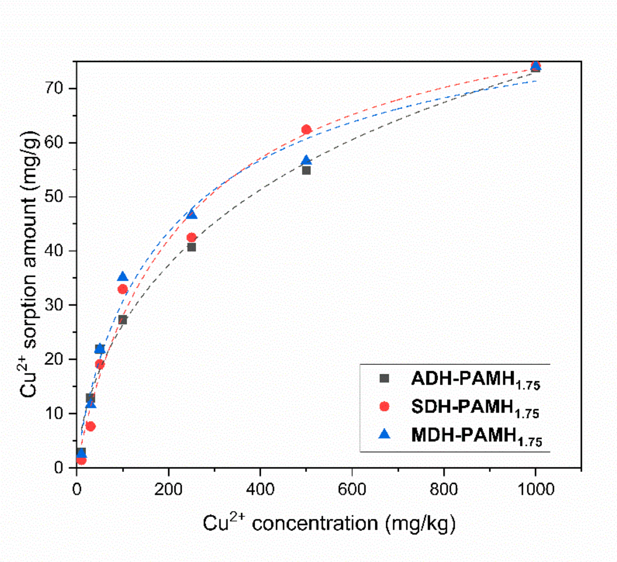

Figure S5. Cu^{2+} Sorption isotherm of ADH-PAMH$_{1.75}$, SDH-PAMH$_{1.75}$, and MDH-PAMH$_{1.75}$.

6. FT-IR spectroscopy for metal-complexd ADH-PAMH1.75

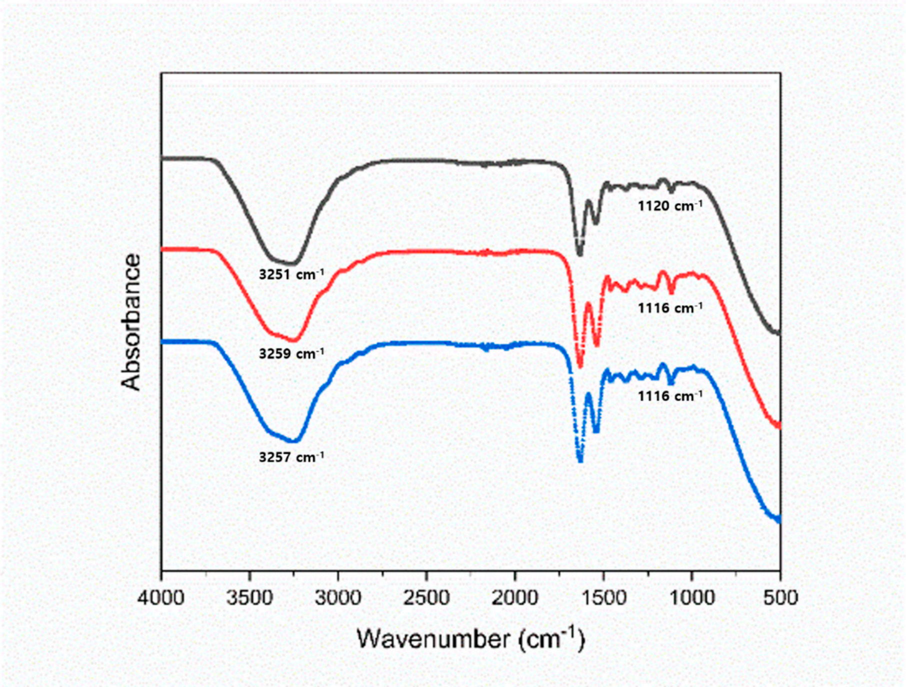

Figure S6. ATR-IR for metal absorbed ADH-PAMH1.75 (black: wet ADH-PAMH1.75, red: Cu^{2+} absorbed ADH-PAMH1.75, blue: Cd^{2+} absorbed ADH-PAMH1.75)
